# Fatal Cryptococcal Meningitis in a Patient with Chronic Lymphocytic Leukemia

**DOI:** 10.4084/MJHID.2012.039

**Published:** 2012-06-14

**Authors:** Oguzhan Sıtkı Dizdar, Faruk Karakeçili, Belkıs Nihan Coşkun, Beyza Ener, Rıdvan Ali, Reşit Mıstık

**Affiliations:** 1 Department of Internal Medicine, Uludag University Medical School, Bursa, Turkey; 2 Departments of Microbiology and Infectious Diseases, Uludag University Medical School, Bursa, Turkey; 3 Division of Hematology, Uludag University Medical School, Bursa, Turkey

## Abstract

Patients with chronic lymphocytic leukemia (CLL) are susceptible to infections, especially opportunistic infections. We have described a patient with CLL who had cryptococcal meningitis. Despite lack of previous immunosuppressive treatment history, the patient experienced serious and fatal fungal infection. Physicians should be alert for a diagnosis of cryptococcal meningitis in patient with CLL who developed fever and headache.

## Introduction

Cryptococcosis is an infection caused by the yeast fungus *Cryptococcus neoformans*. Cryptococcal meningoencephalitis is invariably fatal without appropriate therapy, and most affected patients are immunocompromised. The most common forms of immunosuppression other than HIV include organ transplantation, glucocorticoid therapy, lymphatic malignancies (especially Hodgkin’s lymphoma), and other rare conditions such as sarcoidosis.^1^,^2^

Chronic lymphocytic leukemia (CLL) is associated with impaired humoral and cellular immunity which results in susceptibility to infections. Neutropenia and chemotherapy also contribute to this condition. Herein, we have reported a case of cryptococcal meningitis in a patient with CLL.

## Case Report

A 54-year-old male patient was admitted complaining of fever, coughing, headache and sputum during the previous month. He had a two-week history of oral amoxicillin clavulanate (1 gr, twice daily) and ciprofloxacin (500 mg, twice daily) use. He was diagnosed with stage two chronic lymphocytic leukemia (CLL) one year prior to admission. Chemotherapy or any immunosuppressive treatment including steroids had not been administered to the patient during his follow-ups. He did not have any CLL complications.

A physical examination was performed, and he had a body temperature of 38.5 ºC, a blood pressure of 135/80 mmHg and a regular pulse rate of 100 beats per minute. The pathological findings of the examination included cervical lymphadenopathy and right basal crackles upon auscultation of the lung. A neurological examination revealed a normal, oriented man without neurologic deficits. Laboratory tests revealed a hemoglobin level of 9, 3 g/dl, a white blood cell count of 58.900/mm^3^ (86% lymphocytes) and a platelet count of 159.000 cells/mm^3^. Serum glucose, electrolyte and kidney function tests were normal. A peripheral blood examination demonstrated lymphocyte predominance and the presence of basket cells. Urinalysis and urine and blood cultures were negative for any microorganism.

Cefepime (2 grams, three times daily) was administered intravenous to the patient in addition to oral clarithromycin (500 mg, twice daily). Aspartate aminotransferase (AST) and alanine aminotransferase (ALT) rapidly increased, and we decided to cease clarithromycin on the first day of treatment. The patient’s fever remained on the fourth day of treatment, and we replaced cefepime with vancomycin and carbapenem. Thoracic computed tomography (CT) exhibited parenchymal infiltration. A brain CT scan revealed normal findings. Due to an on-going disturbance in the liver function tests, vancomycin treatment was ceased on the second day. The patient’s condition did not improve with these treatments, and he continued to have temperatures up to 39 C. In the meantime, the patient’s condition was complicated with Coombs positive hemolytic anemia, and daily prednisolone was administered for two weeks (60 mg on the first three days, followed by 30mg/day).

The patient’s headache did not resolve during his hospitalization, and on the 21st day after admission, the patient lost consciousness. While blood cultures from the early period of hospitalization were negative, a blood culture obtained on the 18th day of hospitalization revealed mucoid yeast colonies on the 3rd day following collection. A lumbar puncture sample contained 20 leukocytes/mm^3^ and, 100 erythrocytes/mm^3^, and the protein and glucose levels were 36,2 mg/dl and 26 mg/dl respectively. Encapsulated budding yeast cells resembling *Cryptococcus* were observed using an India ink preparation. The blood cultures were continuously monitored using an automated blood culture system (BACTEC 9000/BD), and Sabouraud dextrose agar (SDA; 4%) and inhibitory mould agar (IMA) were used for CSF culture. Inoculated culture media were evaluated after 72 hours and morphology results from cornmeal–tween 80 agar and the API ID 32C system (bioMerieux-USA) were used for identification. *Cryptococcus neoformans* was identified as the offending pathogen, and it was separated from *Cryptococcus gattii* by growth features on canavanine-glycine-bromothymol blue (CGB) agar. Amphotericin B was started at a dose of 0,8 mg/kg. However, the patient’s condition did not improve, and he died one day after treatment initiation due to respiratory failure and septic shock.

## Discussion

Cryptococcal meningoencephalitis is an important opportunistic infection, and most affected patients have T cell dysfunction. Few cases of cryptococcal meningoencephalitis in patients with CLL have been reported. The predilection for cryptococcal infection in patients with CLL was confirmed in the largest published case series, which consisted of 41 patients with neoplastic disease.^3^ In that series, most patients with CLL (n:5) had been treated with high dose steroids. In most other case reports, CLL patients complicated with cryptococcal infections had been treated with various chemotherapies.

Lumbar puncture with a high opening pressure and careful evaluation of the cerebrospinal fluid (CSF) with India ink and/or cryptococcal antigen testing should suggest the diagnosis of cryptococcal meningoencephalitis in most cases. Although CSF examination with India ink can demonstrate encapsulated yeast forms, a culture nearly always establishes the diagnosis. Low glucose levels and elevated protein levels are frequently observed. Inflammatory cell counts have a lymphocytic predominance. Cryptococcal antigens can also be found in the blood.^4^ Neuroimaging is most often normal, as observed in our patient.

In patients with CLL, the incidence of major infection, especially *Streptococcus pneumoniae*, *Staphylococcus aureus*, *Haemophilus influenzae* and Herpes viruses, increases with disease stage and active treatment. Patients treated with purine analogs (e.g., fludarabine) are at increased risk of cryptococcal infections.^5^ Our case did not have this treatment, which sets it apart from most other cases in the literature. Table 1 shows the recently published characteristics of cryptococcal meningitis in patients with CLL.^6^

The significant clinical and laboratory predictors of treatment failure during initial therapy include high lumbar puncture opening pressure, a low CSF glucose level, a CSF leukocyte count less than 20 cells/μL, a positive India ink examination of the CSF, evidence of disseminated disease by positive blood cultures or high antigen titers in the blood or CSF, abnormal mental status and underlying malignancies.^7^ The nature of the underlying immunosuppression is important for prognosis in HIV-seronegative patients. The prognosis among patients with malignancy is much worse than among those with immunosuppression related to glucocorticoid therapy or those with no apparent risk factors.^8^ Our patient had most of these unfavorable prognostic factors.

Unlike many other publications concerning the coexistence of CLL and cryptococcal infection, our patient had no history of previous immunosuppressive treatment, and the patient was in remission for CLL. However, administering a steroid for Coombs-positive Hemolytic anemia may have contributed to this serious infectious disease.

Patients with CLL are prone to infections due to underlying cellular and humoral immunodeficiencies. However, cryptococcal meningitis should be suspected in any CLL patient with fever and headache, and a lumbar puncture should be performed on these patients.

Treatment options for cryptococcal meningitis include amphotericin B and fluconazole. Once the diagnosis was made, we administered amphotericin B for two days. Unfortunately, because the patient died of disease progression, we were unable to observe the treatment response. A progressive clinical course did not allow us to diagnose a transformation to lymphoma (Richter’s syndrome).

## Conclusion

We presented one CLL patient who developed cryptococcal meningitis even though he was not receiving any immunosuppressive treatment.

## Figures and Tables

**Table 1:** The characteristics of cryptococcal meningitis in different patients with CLL.

References		Age, Gender	Admission symptoms	Previous treatment for CLL	CSF Leukocyte count/ml^3^	CSF Protein mg/dl	CSF Glucose mg/dl	CSF Microscopy and culture§	Blood culture§	Antifungal treatment	Outcome
Reisfeld - Zadok et al	Case 1	78,F	Disorientation, euphoric and somnolent episodes	A combination of cyclophosphamide oncovine and prednisone, and for the previous 6 months intravenous cyclophosphamide (1000 mg) every 4 weeks and prednisone (10 mg a day)	175	161	40	Positive	Negative	Amphotericin B (1 mg/kg) for 2 weeks, subsequently fluconazole	Response to treatment
Reisfeld - Zadok et al	Case 2	82,M	Headache and low- grade fever	Over the years with leukeran	36	186	7	Positive	Negative	Amphotericin B, switched first to the liposomal formulation and later to fluconazole	Response to treatment
*	Present case	55,M	Headache, fever, cough	No, but prednisolone for two weeks (60 mg on first three day, then 30 mg) due to haemolytic anaemia	20	36	26	Positive	Positive	Amphotericin B	Patient died

Abbreviations:

* Present case, M: male, F: female, CSF: cerebro spinal fluid,

§ Negative/positive for *C. neoformans*

## References

[1] Tay ST, Rohani MY, Hoo TS, Hamimah H (2010). Epidemiology of cryptococcosis in Malaysia. Mycoses.

[2] Akcaglar S, Sevgican E, Akalin H, Ener B, Tore O (2007). Two cases of crytococcal meningitis in immunocompromised patients not infected with HIV. Mycoses.

[3] Kaplan MH, Rosen PP, Amstrong D (1977). Cryptococcosis in a cancer hospital: clinical and pathological correlates in forty-six patients. Cancer.

[4] Tsiodras S, Samonis 1G, Keating MJ, Kontoyiannis DP (2000). Infection and immunity in chronic lymphocytic leukemia. Mayo Clin Proc.

[5] Samonis G, Kontoyiannis DP (2001). Infectious complications of purine analog therapy. Curr Opin Infect Dis.

[6] Reisfeld-Zadok S, Elis A, Syzper-Kravitz M, Chowers M, Lishner M (2009). Cryptococcal meningitis in chronic lymphocytic leukemia patients. ISR Med Assoc J.

[7] Diamond RD, Bennet JE (1974). Prognostic factors in cryptococcal meningitis. A study in 111 cases. Ann Intern Med.

[8] Pappas PG, Perfect JR, Cloud GA, Larsen RA, Pankey GA, Lancaster DJ, Henderson H, Kauffman CA, Haas DW, Saccente M, Hamill RJ, Holloway MS, Warren RM, Dismukes WE (2001). Cryptococcosis in human immunodeficiency virus-negative patients in the era of effective azoletherapy. Clin Infect Dis.

